# Increased Drought Tolerance through the Suppression of *ESKMO1* Gene and Overexpression of *CBF*-Related Genes in Arabidopsis

**DOI:** 10.1371/journal.pone.0106509

**Published:** 2014-09-03

**Authors:** Fuhui Xu, Zhixue Liu, Hongyan Xie, Jian Zhu, Juren Zhang, Josef Kraus, Tasja Blaschnig, Reinhard Nehls, Hong Wang

**Affiliations:** 1 School of Life Sciences and Technology, Tongji University, Shanghai, China; 2 KWS SAAT AG, Einbeck, Germany; 3 School of Life Science, Shandong University, Shandong, China; Purdue University, United States of America

## Abstract

Improved drought tolerance is always a highly desired trait for agricultural plants. Significantly increased drought tolerance in *Arabidopsis thaliana* (Columbia-0) has been achieved in our work through the suppression of *ESKMO1 (ESK1)* gene expression with small-interfering RNA (siRNA) and overexpression of *CBF* genes with constitutive gene expression. *ESK1* has been identified as a gene linked to normal development of the plant vascular system, which is assumed directly related to plant drought response. By using siRNA that specifically targets *ESK1*, the gene expression has been reduced and drought tolerance of the plant has been enhanced dramatically in the work. However, the plant response to external abscisic acid application has not been changed. *ICE1*, *CBF1*, and *CBF3* are genes involved in a well-characterized plant stress response pathway, overexpression of them in the plant has demonstrated capable to increase drought tolerance. By overexpression of these genes combining together with suppression of *ESK1* gene, the significant increase of plant drought tolerance has been achieved in comparison to single gene manipulation, although the effect is not in an additive way. Accompanying the increase of drought tolerance via suppression of *ESK1* gene expression, the negative effect has been observed in seeds yield of transgenic plants in normal watering conditions comparing with wide type plant.

## Introduction

Drought stress is a major limiting factor for crop production worldwide [1]. In 2012, a severe drought in the United States caused heavy losses in crop production, especially in corn, and farmers produced less than three-fourths of the corn that the U.S. Department of Agriculture anticipated [2]. In China, around 20 million hectares of land are at risk of drought each year [3]. Globally, estimated crop losses due to water limitation exceed $10 billion annually [4]. Improving yield under drought, therefore, is a continuous challenge for agriculture, especially for modern breeding.

The development of modern plant biotechnology provides new hope for generating crops with increased drought tolerance. Understanding the response of plants to drought stress is the first step for development of stress tolerance plant through plant biotechnology. Gene expression experiments have identified several hundred genes that are either induced or repressed during drought. Some of those genes encode proteins that play important role in protecting cells from dehydration, such as the enzymes required for biosynthesis of various osmoprotectants, late-embryogenesis–abundant (LEA) proteins, antifreeze proteins, chaperones, and detoxification enzymes [5]. Some others are responsible for gene products including transcription factors, protein kinases, and enzymes involved in phosphoinositide metabolism. C-repeat/dehydration–responsive element binding factors (*CBF*s) are AP2/ERF-type transcription factors, which make up a critical gene cluster of the second group. During in the stress condition, *CBF* genes are rapidly induced in response to abiotic stress, such as dehydration and cold [6], [7]. The CBF proteins in turn activate expression of a set of target effector genes by binding to a core sequence in their promoter, C-repeat (CRT) / dehydration response element (DRE) [8], [9], [10], [11]. *CBF* genes appear to be ubiquitous in plant species and almost always present as a gene family [12], [13]. In *Arabidopsis*, the three characterized *CBF* genes are *CBF1*, *CBF2*, and *CBF3*, which are organized in tandem on chromosome 4 [14]. *CBF1* and *CBF3* are positive regulators whereas *CBF2* has a negative regulatory role [15]. *CBF* transcription factor genes are induced by the constitutively expressed inducer of *CBF* expression (*ICE1*) by binding to the *CBF* promoter [16], [17]. The *ICE1*-*CBF* cold response pathway is conserved in diverse plant species [14], [17], [18]. Constitutive overexpression of *CBF* transcription factors in transgenic plants has increased the plant tolerance to freezing, salt, and drought stresses [8], [19], [20], [21], [22], [23]. This functional conservation has suggested the *ICE1*-*CBF* genes are important targets for crop improvement for drought tolerance through genetic engineering [24].

*ESK1* is a newly discovered member of the second group. It was initially identified as conferring freezing tolerance; a significantly high proline content accumulates in *esk1* mutants [25]. The gene product of *ESK1* belongs to an uncharacterized plant-specific protein family containing 48 members [26]. Bioinformatics analysis of genes whose expression modified by the *eskimo1* mutation showed that a large number of genes were previously reported linking to plant response to salt, osmotic stress, and the stress hormone abscisic acid (ABA) [25], [26]. Later work showed that the mutant has a clear advantage in response to drought and salt stress: In standard and drought conditions, transpiration rate of mutant is lower than in wild type (WT) [27]. A biologically relevant parameter is the water required per biomass unit, and with this measure, the *esk1* mutants clearly have shown a higher water use efficiency and photosynthetic rate compared to WT [27]. This higher water use efficiency was independent of stomata closure through ABA biosynthesis. Measurement of root hydraulic conductivity suggests that the *esk1* vegetative apparatus suffers water deficit because of a defect in water transport system [27], [28]. *ESK1* promoter-driven reporter gene expression has been observed in xylem and fibers, the vascular tissue which is responsible for the transport of water and mineral nutrients from the soil to the shoots, via the roots. Moreover, in cross sections of hypocotyls, roots, and stems, collapsed xylem vessel has been observed in *esk1* mutant [28]. The *ESK1* gene, therefore, was inferred to play a major role in whole plant water economy. *ESK1* has homologues in numerous species, and it is reasonable to hypothesize that manipulation of *ESK1* in crops could improve water use efficiency.

With an understanding of the molecular mechanism, several gene manipulation approaches have been employed to increase plant drought tolerance. The manipulated genes include those encoding enzymes required for the biosynthesis of various osmoprotectants or enzymes for modifying membrane lipids, LEA protein, and detoxification enzyme [29], [30], [31]. To date, the *CBF* genes are the most explored genes for improving crop stress tolerance because of the ability of this transcription factor to regulate an entire set of genes in a stress-response pathway [8], [32], [33]. When rice *OsDREB1A* [34] or corn *ZmDREB1A* is constitutively overexpressed in *Arabidopsis*, the downstream target genes regulated by the *Arabidopsis DREB1* (e.g., *RD29A*) are induced, resulting in desiccation tolerance under 15% humidity [35]. In another study, 35S: *CBF1* transgenic tomato plants are more resistant to water-deficit stress by showing less plant wilting and leaf curling than WT controls after 21-d water withdrawal in the same pot [36]. Constitutive overexpression of two wheat *CBF* factors in barley substantially improves survival under severe drought or cold. In addition, expression of *DREB* factors in wheat and barley under the control of drought-inducible promoters allows for normal development, together with significantly improved survival under severe drought [37].

In spite of the extensive evaluation of *CBF* factors, only a few studies have shown a clear improvement in drought tolerance in crops under field conditions [38]. Considering the complexity of the plant stress response, it has been assumed that better drought tolerance might be obtained if multiple genes involved in different stress response pathways could be manipulated together through molecular stacking. In this work, we selected *ICE1* as well as the *CBF1* and *CBF3* genes involved in one stress-activated pathway and the *ESK1* gene from another stress-regulating pathway as targets of gene manipulation. Here, *ICE1* or *CBF1* and *CBF3* genes were overexpressed under the control of a constitutive promoter whereas the *ESK1* gene was suppressed with siRNA technique. Using gene stacking, we combined the two expression cassettes into one transformation vector for *Agrobacterium*-mediated plant transformation. The results obtained by testing the concept in the model plant *Arabidopsis* showed a significant increase in drought tolerance compared to non-transgenics, indicating that multiple gene manipulation might be a promising strategy for improving stress response and especially drought tolerance in plants.

## Results

### Manipulation of target gene expression through knock-down and overexpression

**Figure 1:**
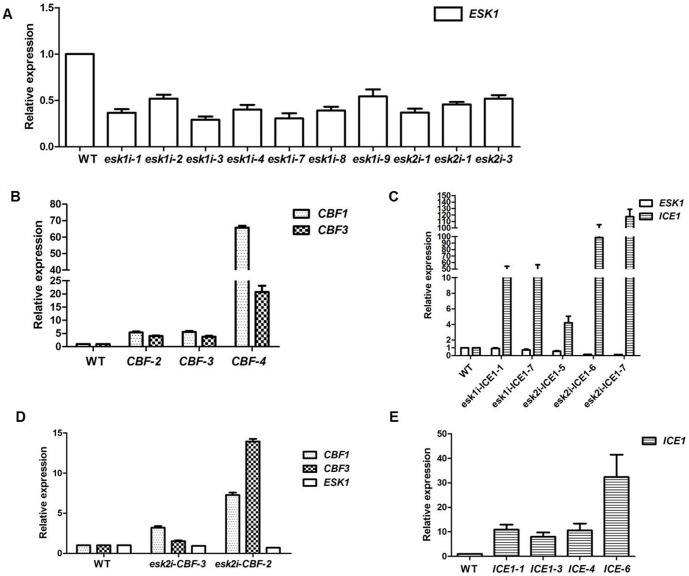
qRT-PCR results show *ESK1*, *ICE1*, *CBF1*, and *CBF3* expression in different transgenic lines. (**A**) *ESK1* was tested in two-week-old *ESKi* transgenic lines. (**B**) *CBF1* and *CBF3* were tested in two-week-old *CBF* transgenic lines. (**C**) *ESK1* and *ICE1* were tested in two-week-old *eski-ICE1* transgenic lines. (**D**) *ESK1*, *CBF1* and *CBF3* were tested in two-week-old *esk2i-CBF* transgenic lines. (**E**) *ICE1* was tested in two-week-old *ICE1* transgenic lines. Values represent means ±SD (error bar) of three replicates.

To knock down gene expression, a siRNA targeting specifically the *ESK1* gene was designed, and to overexpress the desired *ICE1* or *CBF* gene, the 35S promoter was placed in front of the gene for constitutive expression. The gene suppression and overexpression cassettes were integrated into the *Arabidopsis* genome, either alone or combined, via the flora dipping method. In analysis of gene expression by qRT-PCR, the plant transformed with siRNA cassette that suppresses *ESK1* showed clear knock-down of *ESK1* expression. Although the level of suppression varied from line to line, most plants showed about or more than 50% reduction in gene expression (Fig. 1A). In comparison, the transgenic lines derived via using only the pGPTV-*ESKi* vector showed a better suppression of *ESK1* gene expression than those obtained through use of the vector with the stacked overexpression and suppression cassettes (Figs. 1A, C, D).

Analysis of either *ICE1* or *CBF* overexpression showed the transgenic lines with the relevant transgene cassette had significantly higher *ICE1* or *CBF* expression compared to WT (Figs. 1B–E). For further evaluation of the concept, the best-performing plants based on qRT-PCR results were selected and their T2 seeds produced following the protocol described in the Methods part.

### The response of transgenic *Arabidopsis* to osmotic stress *in vitro*

**Figure 2:**
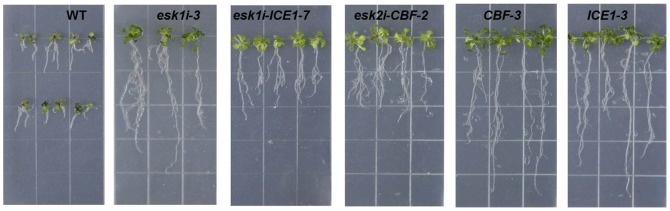
Response of transgenic lines to osmotic stress. Seedlings of different transgenic lines were subjected to osmotic stress: WT, *esk1i*-3, *esk1i*-*ICE1*-7, *esk2i*-*CBF-*2, *CBF*-3, *ICE1*-3. Four-day-old WT or transgenic seedlings were transferred to 1/2 Murashige and Skoog (MS) medium previously infused with 30% PEG for 14 days. Experiments were repeated at least three times with similar results. At least 30 seedlings per genotype were measured in each replicate.

A distinct difference emerged in *in vitro* stress response between transgenic and WT plants. When growing on medium without any applied external stress, the majority of transgenic lines showed an almost identical phenotype to WT plants. However, when growing on medium with 30% polyethylene glycol (PEG), the transgenic plants with suppressed *ESK1* had much better root system growth than WT plants: The main root was longer, and the number of lateral roots was greater (Fig. 2). Leaf growth also differed between the transgenic and WT plants although not as much as the root system: All transgenic lines had eight leaves after 18 days of growth on medium with PEG whereas WT had only six. 15 *ESKi* transgenic lines were tested and the result of response to PEG was similar (data not shown).

Compared to the transgenic lines with the suppressed *ESK1* alone, the plants obtained through using stacking vector that suppress *ESK1* and overexpress either *ICE1* (14 lines) or *CBF* (3 lines) presented a similar performance on medium with PEG. After 14 days of growth with PEG, more and larger leaves were observed in transgenic lines compared to WT (Fig. 2). The growth of the root system had the same pattern with transgenic lines showing less effect from PEG treatment.

The responses to *in vitro* osmotic stress observed from transgenic plants with only the overexpression for *CBF* (5 lines), or *ICE1* (4 lines) were also similar to those with *ESK1* suppressed (Fig. 2). On normal 1/2 MS medium without PEG, no phenotype difference was observed between transgenics and WT control. On PEG medium, however, most transgenic plants clearly showed better growth than control plants after 14 days: The development of the root system from plants with *CBF* or *ICE1* overexpression was more robust, with a longer main root and more lateral roots. Nevertheless, several lines of *ICE1* transgenic plants showed a stress phenotype like that of WT, although the *ICE1* expression in those plants was much higher than in WT.

### The response of transgenic plants to ABA stress *in vitro*

**Figure 3:**
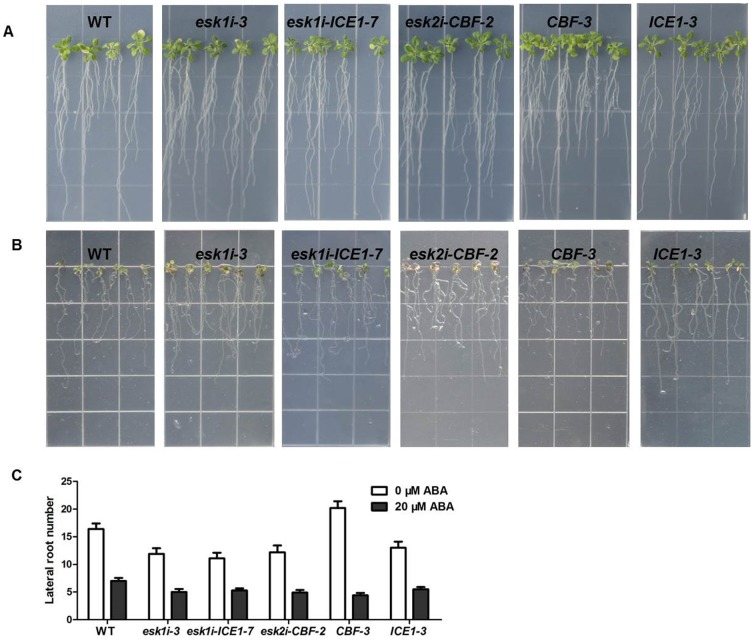
Seedling growth in response to ABA for WT and transgenic plants. (**A**) Four-day-old seedlings grown on 1/2 MS medium were transferred to 1/2 MS medium without ABA. (**B**) Sensitivity of seedlings to ABA. Four-day-old seedlings grown without ABA were transferred to 1/2 MS medium with 20 µM ABA. The photographs were taken 14 d after the transfer. (**C**) Quantification of lateral root number. 30 seedlings were measured in each experiment. Values represent means ±SD (error bar) of three replicates. At least 30 seedlings per genotype were measured in each replicate.

Suppression of *ESK1* gene expression did not significantly influence the sensitivity of plants to external ABA application. On medium without additional ABA, transgenic plants containing *ESK1* suppression cassette appeared almost identical to WT plants in their growth (Fig. 3A). On medium with 20 µM ABA for 14 days, the two still did not differ, and both appeared to suffer the effects of growth on ABA, showing leaves with yellow-brown stress symptoms (Fig. 3B).

**Figure 4:**
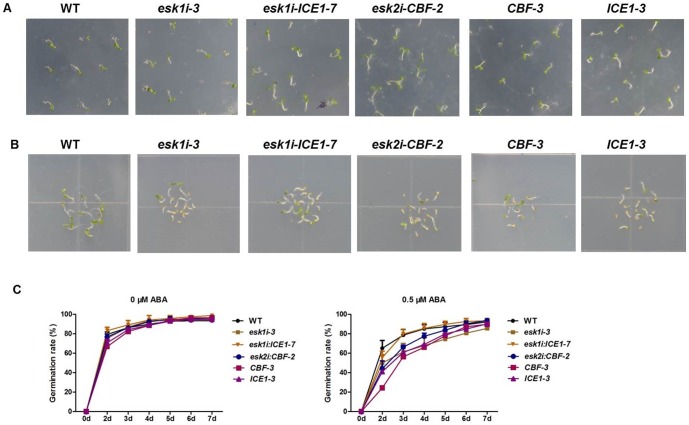
Seed germination in response to ABA for WT and transgenic plants. (**A**) and (**B**) Photographs of young seedlings at 5 d after the end of stratification. Seeds were germinated and allowed to grow on horizontal agar medium containing 0 or 0.5 µM ABA. (**C**) Seed germination time course of the six genotypes grown on medium without ABA or with 0.5 µM ABA. Values represent means ±SD (error bar) of three replicates. At least 100 seeds per genotype were measured in each replicate.

The only difference observed between transgenic lines and WT plants was in seed germination time. On medium containing 0.5 µM ABA (Fig. 4B), the transgenic seeds showed delayed germination compared to WT control whereas on medium without ABA (Fig. 4A), the transgenic seeds germinated at the same time as WT.

Similar results were obtained for plant overexpression of *CBF* or *ICE1* in response to ABA stress *in vitro* (Figs. 3, 4). On medium without ABA, both transgenic and control plants showed similar growth with no abnormal phenotype observed in the majority of plants during the first 18 days of growth.

On medium with 20 µM ABA, both transgenic and WT plants were negatively affected. The elongation of the main root was inhibited, the number of lateral roots was reduced, and the leaves showed yellow-brown stress symptoms. No clear differences between transgenic lines and non-transgenic controls were observed (Fig. 3).

### The performance of greenhouse transgenic plants in response to drought

To mimic most completely the interaction of plants and their natural environment, the transgenic plants showing the potential to increase drought tolerance via the *in vitro* osmotic test were transferred to greenhouse for evaluation of drought tolerance. The testing protocol was established based on the water-loss rate in greenhouse and the survival rate of plants in the protocol of water withdrawal and re-watering. Plants growing 2 weeks after germination in the described conditions were withdrawn from water for 14 days, so that the soil water content reached around 20%, which is considered a serious drought condition [27]. Following this threshold point, the plants were then fully watered and grown under a normal watering program for two more weeks before survival rates were evaluated.

**Figure 5:**
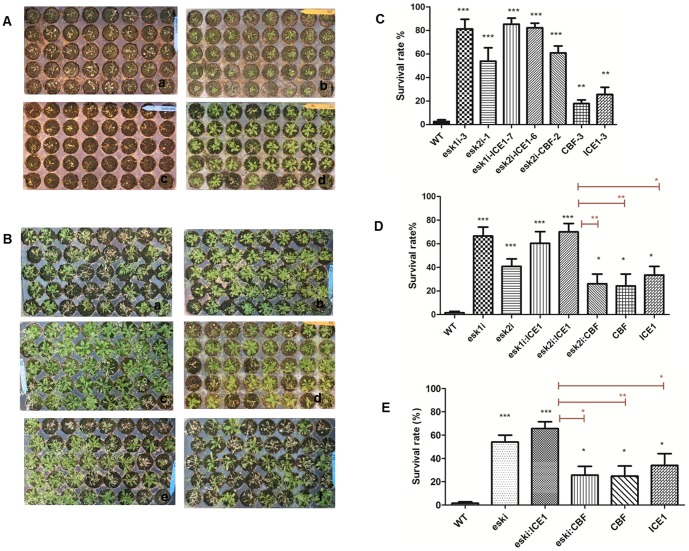
Drought responses of WT and transgenic plants. (**A**) and (**B**) Drought tolerance assay. 7-d seedlings were transferred to soil for another 1 week, subjected to drought by water withholding for 14 d, and then re-watered for 7 d. WT (Aa and Ac), *esk1i*-3 (Ab and Ad), *esk2i*-1 (Ba), *esk1i*-*ICE1*-7 (Bb), *esk2i*-*ICE1*-6 (Bc), *esk2i-CBF-2* (Bd), *CBF*-3 (Be), and *ICE1*-3 (Bf) plants. (**C**) Survival rate of plants from (A) and (B) after re-watering. (**D**) Survival rate of plants from three individual transgenic lines of each vector. (**E**) Survival rate of plants from all transgenic lines as description in methods and materials. SD (error bars) was calculated from results of three independent experiments (n>30 for each experiment). Asterisks indicate significant differences from the corresponding WT values as determined by Student's *t*-tests (*0.01≤P≤0.05, **P≤0.01, ***P≤0.001). Experiments were repeated at least three times with similar results.

Non-transgenic plants usually died completely under this protocol; however, transgenic plants obtained by the vector pGPTV-*ESKi* alone showed dramatic improvement in drought tolerance in contrast to the WT control plant (Fig. 5A). After 14 days without water, the transgenic plants still appeared green although with some level of dryness (Figs. 5A, B). Two weeks after re-watering, many of the transgenic plants had re-gained growth, and the survival rate of the best-performing line *esk1i*-3 reached 80% (Fig. 5C). In comparison, almost all WT plants became yellow and dry after 14 days without water, seldom regaining growth after water restoration (survival rate 1.25%, Fig. 5C).

Transgenic plants with *ICE1* or *CBF* gene overexpression vector alone also showed increased drought tolerance under greenhouse conditions (Figs. 5B–E). After 14 days without watering, the transgenic plants still remained green and recovered soon after the re-watering; the non-transgenic controls did not recover. Among the transgenic lines with various vectors, the line transformed with the *ICE1* overexpression vector showed relatively better drought tolerance than those transformed with *CBF* overexpression vector (Figs. 5C, D, E).

The improved drought tolerance test response under greenhouse conditions was also achieved in transgenic plants containing the combined *ESKi* cassette and overexpression cassette, especially those with the combination of the *ESKi* and *ICE1* cassettes; the highest survival rate was obtained from the line transformed with *ESKi*-*ICE1* stacking vector at 85.25% (Fig. 5C).

To compare the effects of different vectors on improvement of drought tolerance, the transgenic plants were divided into groups according to the vectors used, and the survival rate data was analyzed using *t*-tests. The results showed that the plants performing best were those transformed with *ESKi*-*ICE1* stacking vector; the lines transformed with the *ESKi* vector showed the second-best improvement while improvement was weakest with overexpression of the *CBF* genes (Figs. 5D, E).

### The effect of transgenes on seed biomass

To evaluate potential applications to agriculture, we investigated the effect of transgenes on plant biomass, especially seed mass, under greenhouse conditions, either normal growth conditions or drought test conditions.

**Figure 6:**
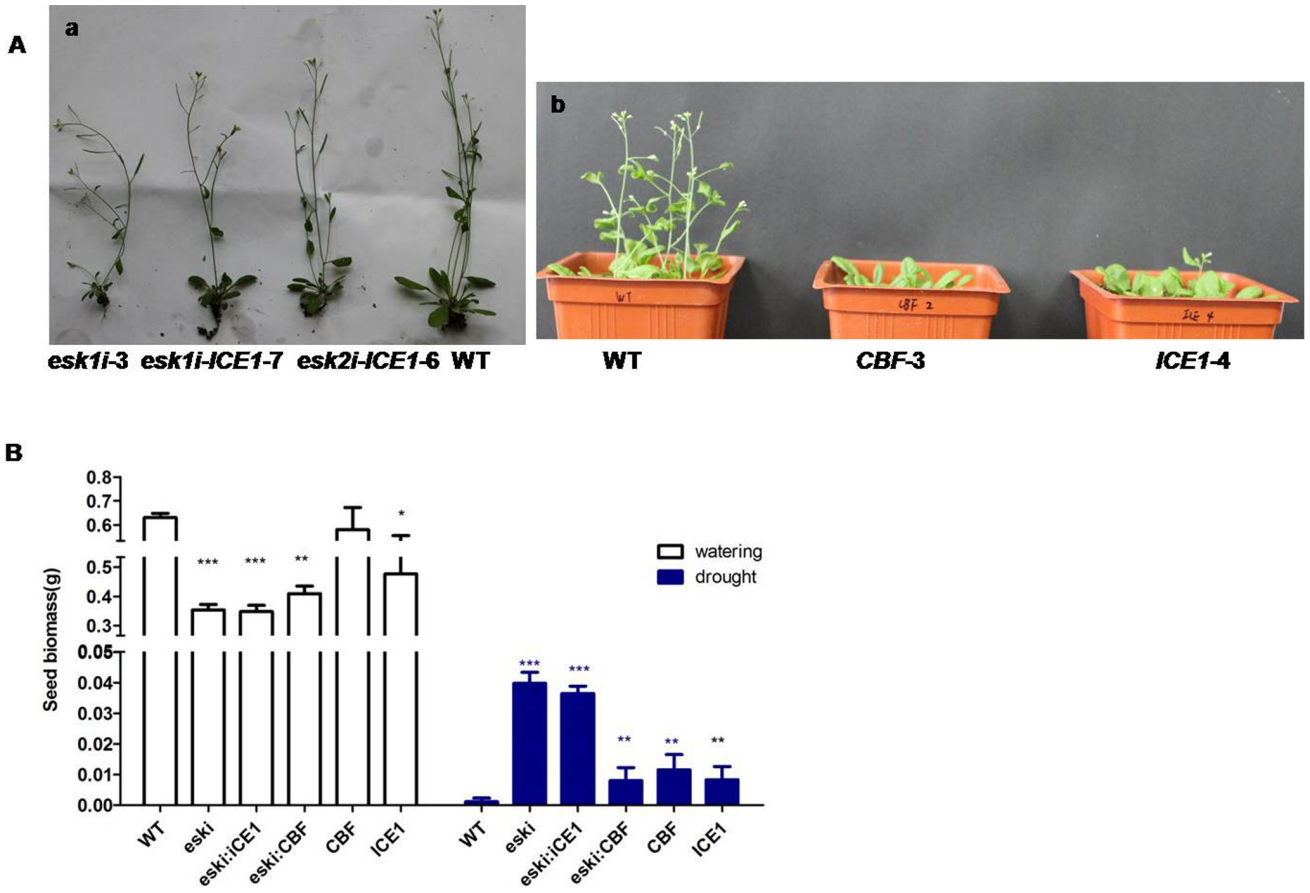
Seed biomass for WT and transgenic plants. (**A**) Phenotype of WT, *esk1i*-3, *esk1i*-*ICE1*-7, *esk2i*-*ICE1*-6, *CBF*-3, and *ICE1*-3 in normal watering environment. a, eight-week-old seedlings, b, six-week-old seedlings. (**B**) Average seed biomass. In the normal watering environment, SD (error bars) calculated from 30 plants of each phenotype and the results of three independent experiments, transgenic lines were as description in methods and materials. After drought treatment, SD (error bars) calculated from 10 plants of each phenotype and the results of three independent experiments, transgenic lines were as description in methods and materials. Asterisks indicate significant differences from the corresponding WT values determined by Student's *t*-tests (*0.01≤P≤0.05, **P≤0.01, ***P≤0.001). Experiments were repeated at least three times with similar results.

Under normal growth conditions in the greenhouse, some *ESK1* siRNA transgenic plants showed a dwarf phenotype with dark leaves (Fig. 6Aa); a delay in flowering was also observed, but most of the plants, otherwise, looked normal. Measuring the mass of seed harvested under normal greenhouse conditions revealed that the *ESK1* siRNA transgenic plants produced less seed than non-transgenic controls. However, upon harvest of the seed from plants that survived the drought test procedure, a clear contrast was evident between the seed yield of transgenic plants and controls, given that most of the non-transgenic plants died of drought. Comparing the seed yield of transgenic plants under drought and normal conditions, a significant reduction was found under drought, with less than 10% of the yield under normal conditions (Fig. 6B).

The impact of transgenes on seed yield showed a similar trend in plants transformed with *ICE1* or *CBF* vector alone and in those transformed with stacking vectors (Fig. 6B). Some dwarf phenotype development was observed during growth under normal conditions whereas in the drought condition, a higher stress tolerance compared to WT was observed, although accompanied by severe seed yield reduction.

## Discussion

Plant genetic engineering has presented great potential for improving the drought tolerance of crops, especially since the discovery of *CBF* genes and their functions in stress tolerance [8], [19], [20], [21], [22], [23]. *ESK1* was recently found to be involved in a stress tolerance pathway that is separate from the *CBF* pathway [25], [26]. The *ESK1* pathway originally was identified as being related to plant freezing tolerance, but Bouchabke-Coussa *et al.* [27] later observed reduced respiration capacity in the *eskl* mutant of *Arabidopsis*, indicating a potential role of *ESK1* in drought tolerance. In our experiment, siRNA specifically targeting *ESK1* was employed to suppress its expression in *Arabidopsis* and the role of *ESK1* was confirmed in drought tolerance: Transgenic plants with efficient *ESK1* expression knock-down showed an obvious increase in osmotic stress tolerance *in vitro* and little effect on root system development by PEG treatment *in vitro* (Fig. 2). Further evaluation confirmed that suppression of the *ESK1* gene significantly enhanced plant drought tolerance (Fig. 5). In ABA *vitro* assay, it has been observed that *ESK1* knock-down transgenic plants has the same sensitivity to ABA change as control (Figs. 3B and 4B), which indicates that acquired stress tolerance of the transgenic plant may not be closely linked to the ABA-regulated network.

The *ESK1* knock-down plants appeared phenotypically normal comparing to non-transgenic plants when grown for 18 days: Plant size, leaf color, and shape were basically same like WT under normal growth conditions (Fig. 3A). Morphological observation also revealed that the number of stomata in the leaves remained unchanged relative to controls (data not shown). The question that arises is how *ESK1* knock-down confers higher tolerance to drought or other stress. One explanation is that the disruption in *ESK1* function may block the normal development of the vascular system. Lefebvre *et al.* [28] observed a kind of collapsed xylem structure in cross sections of hypocotyls, roots, and stems of an *Arabidopsis esk1* mutant. In the current study, we also observed that the vascular system appeared abnormal in both leaf and stem (data not shown). These observations may explain why the respiration rate of a transgenic plant is lower compared to WT and why drought tolerance is higher than in controls.

Measuring the weight of total seeds showed that knocking down the *ESK1* gene can negatively affect seed production under normal growth conditions but that under the drought condition, the yield is much less affected, in contrast to complete loss in the non-transgenic control (Fig. 6B). Linking the yield penalty and the vascular distortion of the *ESK1* knock-down plant, we can infer that manipulation of *ESK1* gene expression can indeed increase the drought tolerance of plants, but expecting no reduction in biomass is not realistic considering the physical distortion of the vascular structure. Adoption of an inducible promoter or tissue-specific promoter may alleviate some negative consequences, but the enhancement of drought tolerance may be compromised at the same time. Therefore, use of *ESK1* for stress tolerance should be carefully balanced. One potential application may be in improvement of ornamental plants, for which biomass is not important.

Many studies have illustrated the potential of manipulating *CBF/DREB* genes to confer improved drought tolerance [20], [21], [37], [39]. For example, overexpression of *CBF1/DREB1B* from *Arabidopsis* improves tolerance to water-deficit stress in tomato, but few plants clearly show enhanced drought tolerance under natural conditions [36]. We speculate that the complexity of the stress response pathway could be the reason and that modification of a single gene in a complicated pathway might not be sufficient to alter plant drought tolerance dramatically. Gene discovery and functional genomics projects have revealed many mechanisms and gene families that confer improved adaptation to abiotic stresses. These gene families can be manipulated into novel combinations, expressed ectopically, or transferred to species in which they do not naturally occur or vary [40], [41], [42]. Therefore, we have designed a gene stacking strategy by combining manipulation of the *CBF* and *ESK1* genes, hoping to obtain at least an additive effect on drought tolerance.

The result showed that gene stacking can indeed further improve drought tolerance in *Arabidopsis*, but an additive effect was not observed. One possibility for the lack of additive effect is that genes stacked in the same transformation vector may not work as efficiently as those in completely independent transformation vectors. qRT-PCR analysis in our work showed that the suppression effect of siRNA targeting *ESK1* was less significant when the same vector was used for the stacked RNAi cassette and the overexpression cassette. In the future, the effect of combined gene manipulation will be evaluated through stacking the gene cassette by crossing the transgenic lines with individual vectors.

## Materials and Methods

### Plant materials and growth conditions

*Arabidopsis thaliana* ecotype Columbia (Col-0) and transgenic plants in Col-0 background were grown under long-day conditions (16 h light/8 h dark) with light intensity at 100 µE m^−2^ s^−1^.

For plate-grown plants, *Arabidopsis thaliana* seeds were surface sterilized with 20% (v/v) bleach and sown on medium containing half-strength Murashige and Skoog (MS) salts [43], 1% sucrose, and 0.8% agar. After stratification for 3 d at 4°C, the plates were kept in a growth incubator under a long-day photoperiod (16 h light, 8 h darkness) at 24°C for 10 d. For transgenic seeds, medium was supplemented with 50 µg/mL kanamycin sulfate.

### Gene isolation and binary vector construct

The leaf of 2-week-old *Arabidopsis* Col-0 was used for DNA and RNA extraction. The Col-0 cDNA sequences were obtained from GenBank (http://www.ncbi.nlm.nih.gov/). Total DNA was extracted from the samples using the DNeasy Plant Mini Kit (Qiagen, Germany), and total RNA was extracted using the RNeasy Plant Kit (Qiagen, Germany). cDNA was synthesized as described in the Thermo Scientific protocol (#K1631).

The *AtCBF1* (AT4G25490) and *AtCBF3* (AT4G25480) were amplified from *Arabidopsis* Col-0 DNA by PCR. *AtCBF1* was amplified with a forward primer containing a BamHI restriction site at the 5′ end (5′-CGGGATCCCTCTGATCAATGAACTCATT-3′) and a reverse primer containing a SacI site at the 3′ end (5′-GCGAGCTCTTAGTAACTCCAAAGCGACA-3′). *AtCBF3* was amplified with a forward primer containing an ApaI site at the 5′ end (5′-GGGCCCGATCAATGAACTCATTTTCTGC-3′) and a reverse primer containing a XbaI site at the 3′ end (5′-GCTCTAGATTAATAACTCCATAACGATACGTCG-3′).

The *AtICE1* (AT3G26744) and *AtESK1* (AT3G55990) cDNA fragments were amplified from *Arabidopsis* Col-0 RNA by RT-PCR. *AtICE1* was amplified with a forward primer containing a XhoI site at the 5′ end (5′-GCCTCGAGGCGATGGGTCTTGACGGAAACAATGGTG-3′) and a reverse primer containing a XbaI site at the 3′ end (5′-GCTCTAGATCAGATCATACCAGCATACCCTGCTGTATCG-3′).

For the RNA interference (RNAi) construct, for a 341-bp specific fragment of *ESK1*, *esk1i*, the sense fragment was amplified by PCR using a forward primer (5′-GCCTCGAGTTGCTAGCATGTCTCCTCTT-3′) and a reverse primer (5′-GCGAGCTCATTCCACGTGTCAGGTAAAC-3′), as was the antisense fragment (forward primer: 5′-CGCCCGGGATTCCACGTGTCAGGTAAAC-3′; reverse: 5′-CGGTCGACTTGCTAGCATGTCTCCTCTT-3′). For a 284-bp specific fragment of *ESK1*, *esk2i*, the sense fragment also was amplified using forward and reverse primers (5′-GCCTCGAGTCAAGTGTGCATTAGAGACG-3′ and 5′-GCGAGCTCATTCCACGTGTCAGGTAAC-3′, respectively), as was the antisense fragment (forward: 5′-CGCCCGGGATTCCACGTGTCAGGTAAAC-3′; reverse: 5′-CGGTCGACTCAAGTGTGCATTAGAGACG-3′). The PCR products were sequenced to ensure that they encoded the expected gene products.

The pGPTV-SdaI vector was used to construct a binary expression vector. The pRNAi-vector was used to construct an intron-spliced hairpin RNA (RNAi construct), and the isolated gene or gene fragment was constructed into the relevant vector to yield pGPTV-*CBF1*-*CBF3* (*CBF*), pGPTV-*ICE1* (*ICE1*), pGPTV-*esk1i* (*esk1i*), pGPTV-*esk2i* (*esk2i*), pGPTV-*esk2i*-*CBF1*-*CBF3* (*esk2i-CBF*), pGPTV-*esk1i*-*ICE1* (*esk1i-ICE1*), and pGPTV-*esk2i*-*ICE1* (*esk2i-ICE1*). The transcription of each gene or gene fragment was under the control of the 35S promoter and 35S terminator.

### *Agrobacterium*-mediated gene transformation

The floral dip method [44] was applied to stably transform the *Arabidopsis* plant by using the *Agrobacterium* strain ATHV containing the designed binary vector. Seeds from treated plants were germinated on media containing 1/2 MS salts, 1% sucrose, 0.8% agar and 50 µg/mL kanamycin. Resistant seedlings were transferred to soil and grown under 16 h light /8 h dark at 24°C in a growth chamber. After PCR confirmation, positive seedlings were used to produce T2 and T3 seeds, which were always subjected to kanamycin selection and PCR confirmation; only the positive plants were used for seed production of the next generation. The kanamycin-tolerant and PCR-positive T3 or T4 plants were used in all experiments. Ecotype Col-0 served as control.

### Quantitative real-time PCR (qRT-PCR)

Total RNA from different *Arabidopsis* plants was isolated using the RNeasy plant mini kit (Qiagen, Germany) according to the manufacturer's instructions. To eliminate any residual genomic DNA, total RNA was treated with ribonuclease-free DNase I (Thermo Scientific). Two micrograms of the total RNA was used as template to synthesize cDNA employing the RevertAid H Minus First Strand cDNA Synthesis Kit (Thermo Scientific). Real-time PCR was performed according to Kant *et al.* [45]. The PCR reaction was performed with three replicates and repeated with three biological samples. Relative quantification values for each target gene were calculated by the 2 (-Delta Delta C(T)) method [46]. For normalizing the amount of total RNA in all *Arabidopsis* samples, *Actin2* (AT3G18780) and *beta tubulin* (AT5G23860) were used as internal reference genes to compare data from different PCR runs or cDNA samples. The GenScript online tool was used to design the primers (https://www.genscript.com/), and primers used in q-PCR are listed in Supplemental Table S1.

### *In vitro* assay for plant response to ABA and PEG

For germination, seeds were planted on the plate containing 1/2 MS salts, 1% sucrose, and 0.8% agar with 0 or 0.5 µM ABA as indicated. Plates were chilled at 4°C in the dark for 3 d and moved to 24°C with a 16 h light/8 h dark cycle. The percentage of seed germination was scored at the indicated times. Germination was defined as an obvious emergence of the radicle through the seed coat [47].

To study the inhibition effect of ABA in post-germinative growth, seeds were sown on 1/2 MS medium with 50 µg/mL kanamycin for 4 d after 3 d stratification, then transferred to 1/2 MS medium containing 0 or 20 µM ABA.

For response to *in vitro* dehydration, 3-day-old seedlings were transferred to 1/2 MS medium previously infused with 30% PEG8000 for 14 d [48]. After growing for 14 d on the treatment medium vertically, seedlings were photographed with a digital camera.

15 *ESKi* transgenic lines, 14 *eski-ICE1* transgenic lines, 3 *eski-CBF* transgenic lines, 5 *CBF* transgenic lines and 5 *ICE1* transgenic lines were used in both PEG and ABA *in vitro* assay. For each treatment, 30 plants of each line have been subjected to the treatment. Each test has been repeated three times. The influence of the tested element was concluded based the result pooled together from three repeats.

### Greenhouse test for drought tolerance

15 *ESKi* transgenic lines, 14 *eski-ICE1* transgenic lines, 3 *eski-CBF* transgenic lines, 5 *CBF* transgenic lines and 5 *ICE1* transgenic lines, which showed desired gene manipulation result in qRT-PCR, were used for drought tolerance test. All seeds from transgenic lines and Col-0 together were sown in 40 5 cm×5 cm pots. Plant growth conditions were as described [49]. All seeds were cold-treated (4°C) for 3 d immediately after planting to ensure uniform germination. Plants were grown in controlled environment chambers at 24°C under sunshine illumination or supplementary illumination from cool-white fluorescent lights (100–150 µmol m^−2^ s^−1^) and irrigated with water three times a week, ensuring soil saturation; the last watering was on day 14 after germination. Two-week-old seedlings were used for drought treatment, in which watering was withdrawn for two weeks. Afterwards, all plants were re-watered according to the pre-drought treatment. Tolerance to drought was determined by the capacity of plants to resume growth after 7 d of re-watering. The test was repeated at least three times. The data obtained was subjected to statistical analysis described as below.

### Measurement of drought effect on seed biomass

14 *ESKi* transgenic lines, 12 *eski-ICE1* transgenic lines, 3 *eski-CBF* transgenic lines, 4 *CBF* transgenic lines and 3 *ICE1* transgenic lines were used for the test. At least 30 plants from each transgenic line and wild type were exposed to both control and drought treatment described as above. After the treatment, the survived plants were grown in normal water condition for seed setting. The seeds were harvested by bag that had been covered to plant in reproductive period. The harvested seeds were cleaned and then dried at room temperature. The weight of seed was measured after 2 weeks drying. The average weight (total harvested seed divided by the number of tested plant) was used as result to compare the drought effect on each line of plant. The data was analyzed based the statistical methods described below.

### Statistics

The results shown are representative of three independent experiments, and within each experiment, treatments were replicated three times. Data were statistically analyzed using SigmaPlot 10.0 and GraphPad Prism 5.

## Supporting Information

Table S1 **Primers used in molecular analyses.**
(DOCX)
