# Supplementary material for: Increased Drought Tolerance through the Suppression of ESKMO1 Gene and Overexpression of CBF-Related Genes in Arabidopsis
Source: PLoS One. 2014 Sep 3;9(9):e106509. doi: 10.1371/journal.pone.0106509 (PMC4153627; doi:10.1371/journal.pone.0106509)
Supplement: Table S1 — Primers used in molecular analyses. (DOCX) [file pone.0106509.s001.docx]

**Supporting Information**

Table S1 Primers used in molecular analyses.

| ***ESK1*-f (AT3G55990)** | TCAGAACTGGAGATGGCAAC |
| --- | --- |
| ***ESK1*-r** | ACCATTGATTCCCATTGGTT |
| ***ICE1*-f (AT3G26744)** | GCACAGCCTACTCTGTTCCA |
| ***ICE1*-r** | CCAGAAACCTCAATCCCAGT |
| ***CBF1*-f (AT4G25490)** | TGAGACGTGTGATACGACGA |
| ***CBF1*-r** | TCCCAAACATTGTCTCCTCA |
| ***CBF3*-f (AT4G25480)** | TCCCTTCTGCCATATTAGCC |
| ***CBF3*-r** | TATTTACACGGCGGAACAGA |
| ***ACT2*-f (AT3G18780)** | CCTCAAAGACCAGCTCTTCC |
| ***ACT2*-r** | CAAGACTTCTGGGCATCTGA |
| ***Tublin*-f (AT5G23860)** | GCACGAAAGAAGTTGACGAA |
| ***Tublin*-r** | TTTCAGACCTGTTGGTGGAA |
